# Multi-Granularity Distillation of Intervention Sensitivity Representations for Multimodal Large Language Models

**DOI:** 10.3390/s26216963

**Published:** 2026-11-04

**Authors:** Zhiguo Zhang, Wei Sun

**Affiliations:** School of Aerospace Science and Technology, Xidian University, Xi’an 710071, China; zhiguozhang95@163.com

**Keywords:** multimodal large language models, knowledge distillation, causal attribution, vision-language models, model compression, UAV object detection

## Abstract

Existing knowledge distillation methods for multimodal large language models (MLLMs) mainly align output distributions or intermediate representations, but rarely supervise how predictions depend on local visual evidence. This limitation may hinder the transfer of fine-grained perception and compositional reasoning. We propose Causal and Multi-source Visual Attribution Distillation (CMVAD), which transfers teacher-derived visual evidence during distillation. Region-level interventions first estimate token-level causal visual attributions, identifying image regions that influence each generated token. The Causal Evidence Adapter Fetch (CEAF) module jointly models token importance and semantic interactions to adaptively aggregate multi-source causal evidence into a unified representation. A Multi-granularity Attribution Distillation (MAD) objective transfers this representation via global evidence distribution alignment, critical-region alignment, and evidence-ranking consistency. Experiments on general visual question answering and compositional reasoning benchmarks show that CMVAD outperforms existing MLLM distillation methods, achieving larger gains in compositional reasoning while retaining competitive visual question answering performance. Results across teacher–student architectures demonstrate its generalizability. We further evaluate VisDrone UAV object detection through overall comparisons, providing supplementary evidence of CMVAD’s applicability to object recognition and localization in UAV imagery.

## 1. Introduction

Multimodal large language models (MLLMs) combine visual encoders with language models to support tasks such as visual question answering (VQA), image captioning, and visual reasoning [1,2]. Their computational and memory requirements, however, remain a barrier to deployment on resource-constrained platforms. Knowledge distillation (KD) addresses this problem by transferring knowledge from a larger teacher model to a compact student model [3,4]. Preserving visual information during this transfer is particularly relevant to UAV imagery, where target recognition and localization often depend on small image regions embedded in cluttered backgrounds. These applications motivate the study of how compact models acquire and retain task-relevant visual knowledge.

**Figure 1:**
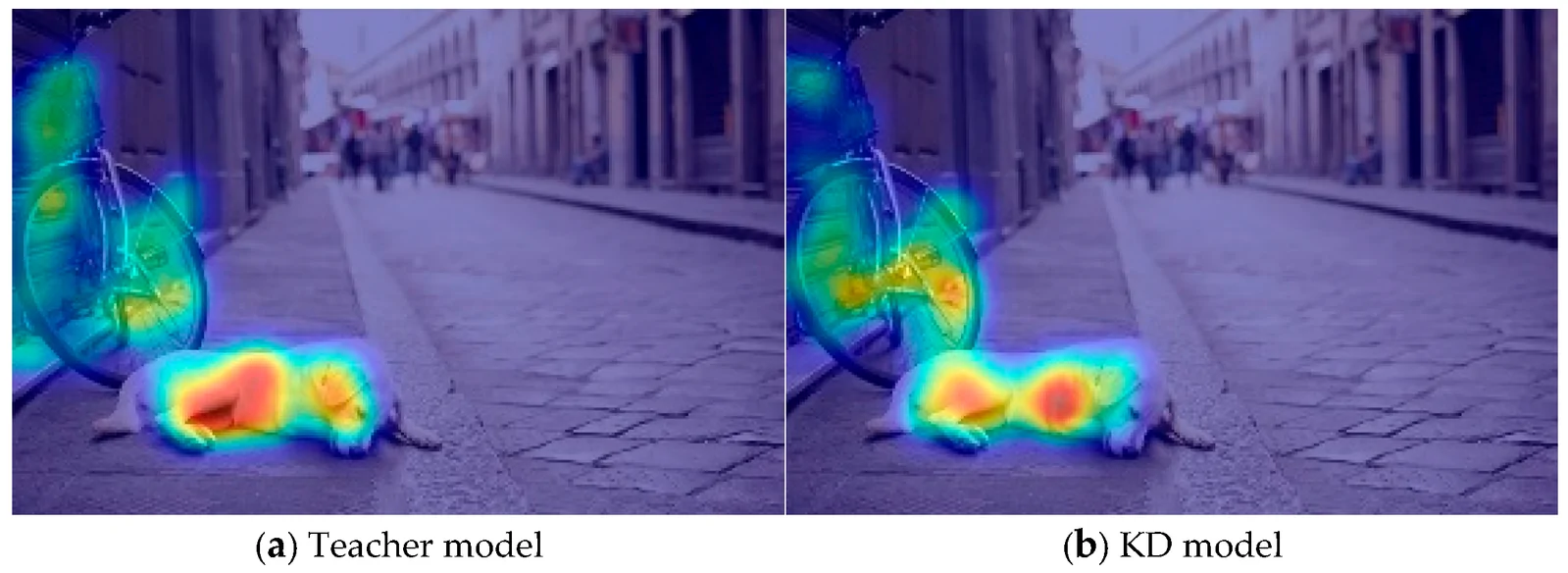
Visual evidence mismatch. The existing distillation methods have matching defects in visual evidence.

Existing multimodal distillation methods align teacher and student output distributions, visual representations, attention patterns, or cross-modal interactions [4,5,6,7]. Despite improvements on conventional VQA benchmarks, these forms of alignment do not directly constrain which image regions causally affect a prediction. As illustrated in Figure 1, a student may reproduce the teacher’s answer while relying on different image regions or language priors. This distinction matters for compositional and spatial reasoning, which require the model to associate objects with their attributes and relationships [8,9]. It also motivates examining visual knowledge transfer in UAV object detection, where recognizing a category must be accompanied by locating the corresponding object. We therefore investigate whether supervision derived from the teacher’s dependence on local visual information can complement conventional distillation objectives.

We propose Causal and Multi-source Visual Attribution Distillation (CMVAD), a framework that uses teacher-derived visual evidence to supervise student learning. First, region-level interventions estimate token-wise visual attributions by measuring changes in the teacher’s prediction scores when local image information is removed or replaced under a fixed textual context. The Causal Evidence Adapter Fetch (CEAF) module then aggregates these attributions through token-importance weighting and pairwise semantic interactions. The resulting evidence representation is transferred using a Multi-granularity Attribution Distillation (MAD) objective, which combines global distribution alignment, critical-region supervision, and ranking consistency. We evaluate CMVAD primarily on general VQA and compositional reasoning benchmarks. As a supplementary application study, we extend the evaluation to UAV object detection on VisDrone, comparing overall and category-wise detection performance. This additional evaluation examines the framework’s applicability to object recognition and localization in aerial imagery.

Experimental results demonstrate that our method effectively balances accuracy and computational complexity, offering substantial advantages over existing methods. The major contributions of this work are fourfold:

• We propose a Causal and Multi-source Visual Attribution Distillation (CMVAD) framework for multimodal large language models, which introduces decision-relevant visual evidence into the knowledge distillation process. Unlike existing multimodal distillation methods that primarily rely on output distributions, intermediate feature representations, or attention maps, the proposed framework explicitly transfers the visual evidence underlying the teacher model’s reasoning process. By extending conventional result-level alignment to visual evidence transfer, CMVAD enables the student model to acquire not only prediction consistency but also the teacher’s visual reasoning mechanism, providing a new paradigm for distilling complex visual reasoning capabilities.
• We develop a Causal Evidence Adapter Fetch (CEAF) module to construct reliable and transferable visual evidence representations. Specifically, CEAF first estimates token-level causal visual attributions through region-level interventions that quantify the causal contribution of image regions to prediction outcomes. It then adaptively aggregates multi-source visual evidence by jointly modeling token importance and semantic interactions among textual tokens, yielding a unified visual evidence representation with enhanced robustness and semantic consistency. Experimental results demonstrate that incorporating CEAF alone improves the accuracy on SugarCrepe from 76.3% to 81.7%, confirming the effectiveness of causal visual evidence modeling.
• We introduce a Multi-granularity Attribution Distillation (MAD) strategy to hierarchically transfer the teacher model’s visual reasoning knowledge. MAD supervises the student model from three complementary perspectives, including global attribution distribution, key decision regions, and attribution ranking consistency, thereby preserving both the spatial distribution and the relative importance of visual evidence. Extensive experiments show that, with a student model of approximately 2B parameters, the proposed CMVAD framework achieves an average accuracy of 63.8% on general VQA benchmarks, outperforming LLaVA-KD-2B by 2.3 percentage points, while reaching 83.6% on SugarCrepe, representing an improvement of 8.3 percentage points. Moreover, CMVAD consistently demonstrates strong generalization and scalability across different teacher-student model configurations.
• We conduct a supplementary evaluation on UAV object detection through comparative experiments and qualitative analysis on VisDrone to assess the applicability of CMVAD beyond multimodal understanding. CMVAD achieves (62.00 ± 0.70%) mAP@0.50 and (39.00 ± 0.36%) mAP@[0.50:0.95]. These results provide empirical support for the application of the proposed distillation framework to UAV object detection.

## 2. Related Work

### 2.1. Multimodal Knowledge Distillation

Knowledge distillation (KD) trains a compact student model using supervision from a larger teacher. The formulation popularized by Hinton et al. [3] transfers information through softened output distributions. Subsequent methods extend this supervision to relationships between representations [10] or decouple the target and non-target components of logit transfer [11]. For MLLMs, distillation must additionally account for visual–language interactions and differences in visual-token resolution between teacher and student architectures [12].

LLaVA-KD [4] combines multimodal output distribution matching with relation distillation to transfer correlations among visual features. Its staged training procedure incorporates distillation into both pre-training and fine-tuning. CompoDistill [6] further aligns teacher and student visual attention to improve visual perception and compositional reasoning.

The selection of teacher supervision is also relevant to UAV object detection, where small foreground regions occupy a limited fraction of images dominated by background content. Du et al. [13] propose Centerness-based Instance-aware Distillation (CID) with Lightweight Mutual Lifting (Light-ML). Their method weights informative regions around object instances and improves the use of classification and localization supervision, with evaluations on VisDrone, UAVDT, and COCO. Chen et al. [14] introduce FFKD-Net, which combines multi-scale feature fusion with hierarchical knowledge distillation to improve lightweight small-object detectors. Experiments on VisDrone and DIOR assess its detection performance and computational efficiency. These studies highlight the role of spatially selective and multi-level supervision in aerial object detection, although their distillation objectives differ from token-level supervision in MLLMs.

Recent studies have examined the use of Transformer architectures and large language models in UAV applications. Kheddar [15] summarizes their applications in aerial perception, object detection, autonomous decision-making, and navigation, while emphasizing the practical constraints imposed by limited onboard computation and energy resources. These observations are closely related to the VisDrone evaluation conducted in this study. Nevertheless, most existing UAV-oriented studies focus on network design, task performance, or deployment optimization. They do not explicitly investigate how decision-relevant visual evidence can be identified by a large multimodal teacher and transferred to a compact student model. CMVAD addresses this unresolved issue by introducing causal visual evidence as an intermediate supervision signal while preserving the original student architecture.

Parameter-level compression offers a complementary strategy for reducing the deployment cost of Transformer-based models. Liu [16] introduces a low-rank importance estimation method that uses the gradients of low-rank adaptation matrices to identify less important parameters and reduce the computational burden of pruning and subsequent fine-tuning. Their method primarily modifies the parameter structure of the model to improve resource efficiency. In contrast, CMVAD does not alter the student architecture or perform explicit parameter pruning; instead, it transfers the visual evidence that is most relevant to the teacher’s predictions. Therefore, pruning and evidence-aware distillation address different aspects of model compression and can potentially be combined to achieve both lower resource consumption and more faithful visual reasoning.

### 2.2. Visual Representation Alignment and Cross-Modal Consistency Modeling

To overcome the limitations of conventional knowledge distillation in modeling visual information, recent studies have increasingly focused on visual representation alignment and cross-modal consistency learning. Existing research has shown that vision-language models optimized primarily with language supervision during visual instruction tuning tend to overlook fine-grained visual semantics, leading to degraded performance on complex visual understanding and reasoning tasks. Consequently, explicitly enhancing the quality of visual representations has become an important direction for improving multimodal learning.

Representative studies have explored visual representation alignment from different perspectives. VIRAL [17] uses pretrained vision foundation models to explicitly align the internal visual representations of multimodal large language models, thereby preserving fine-grained visual details and improving vision-centric reasoning. Large-scale vision–language pretraining models such as CLIP [18] and ALIGN [19] likewise provide aligned visual and textual representation spaces that can serve as visual priors for multimodal learning.

Visual alignment has also been extended to domain-specific applications. MedAlign [20] transfers visual representation and attention knowledge from a domain-specific CLIP model to medical large vision–language models through spatial-aware and attention-aware distillation. Post hoc attribution methods such as Grad-CAM [21] and RISE [22] provide region-level importance maps for explaining model predictions; however, they were developed for explanation rather than direct teacher–student alignment.

Although these methods have substantially improved the quality of visual representations, most of them rely on feature similarity or attention consistency as supervisory signals. Such objectives primarily enforce representational alignment while failing to explicitly characterize the causal contribution of visual regions to the model’s decision-making process. Consequently, the transferred knowledge may preserve feature consistency without faithfully conveying the teacher model’s underlying visual reasoning evidence, leaving considerable room for improving evidence-aware multimodal knowledge distillation.

### 2.3. Interaction Modeling and Process-Level Distillation

As the reasoning capability of multimodal large language models continues to improve, researchers have increasingly recognized that merely aligning prediction outputs or intermediate feature representations is insufficient for effectively transferring the teacher model’s reasoning ability. Consequently, recent studies have shifted their attention from static knowledge alignment toward process-aware knowledge distillation, aiming to preserve the dynamic reasoning behaviors underlying multimodal decision-making.

Visual hallucination mitigation represents another closely related line of research in multimodal large language models. Yang [23] proposes a retrieve-then-compare strategy that retrieves semantically related images and compares the corresponding confidence scores to reduce visually unsupported responses during inference. This approach improves the grounding of an existing MLLM at prediction time, whereas CMVAD operates during the distillation process. Specifically, CMVAD uses region-level interventions to estimate the causal contribution of visual regions and transfers this information from the teacher to the student through multi-granularity attribution supervision. Thus, the two approaches are complementary: retrieval-based decoding corrects hallucinations at inference time, while CMVAD encourages the student to learn a more reliable dependence on decision-critical visual evidence.

Representative studies have explored process-level knowledge transfer from different perspectives. Align-TI [7] explicitly aligns vision–instruction token interactions and intra-response transition probabilities, enabling the student to imitate how the teacher extracts instruction-relevant visual information and generates coherent responses. SDRT [24] uses diverse reasoning-guided responses for self-distillation, allowing a vision–language model to internalize multiple reasoning traces.

Related work in language-model compression also shows the value of rationale supervision. Distilling Step-by-Step [25] trains smaller models with teacher-generated rationales as an additional supervision signal and reports improved data efficiency. Together, these studies motivate moving beyond static output matching toward supervision that captures intermediate interactions or reasoning signals. They do not, however, directly transfer region-level visual evidence defined by prediction changes under controlled image interventions.

These advances indicate that multimodal knowledge distillation is evolving from output-level alignment toward process-level knowledge transfer, with increasing emphasis on modeling the internal reasoning mechanisms of teacher models. Nevertheless, existing approaches primarily focus on interaction patterns or reasoning trajectories, while paying limited attention to the decision-relevant visual evidence that directly supports multimodal reasoning. Consequently, the causal contribution of visual regions remains insufficiently modeled during the distillation process, leaving considerable room for improving evidence-aware knowledge transfer in multimodal large language models.

## 3. Method

**Figure 2:**
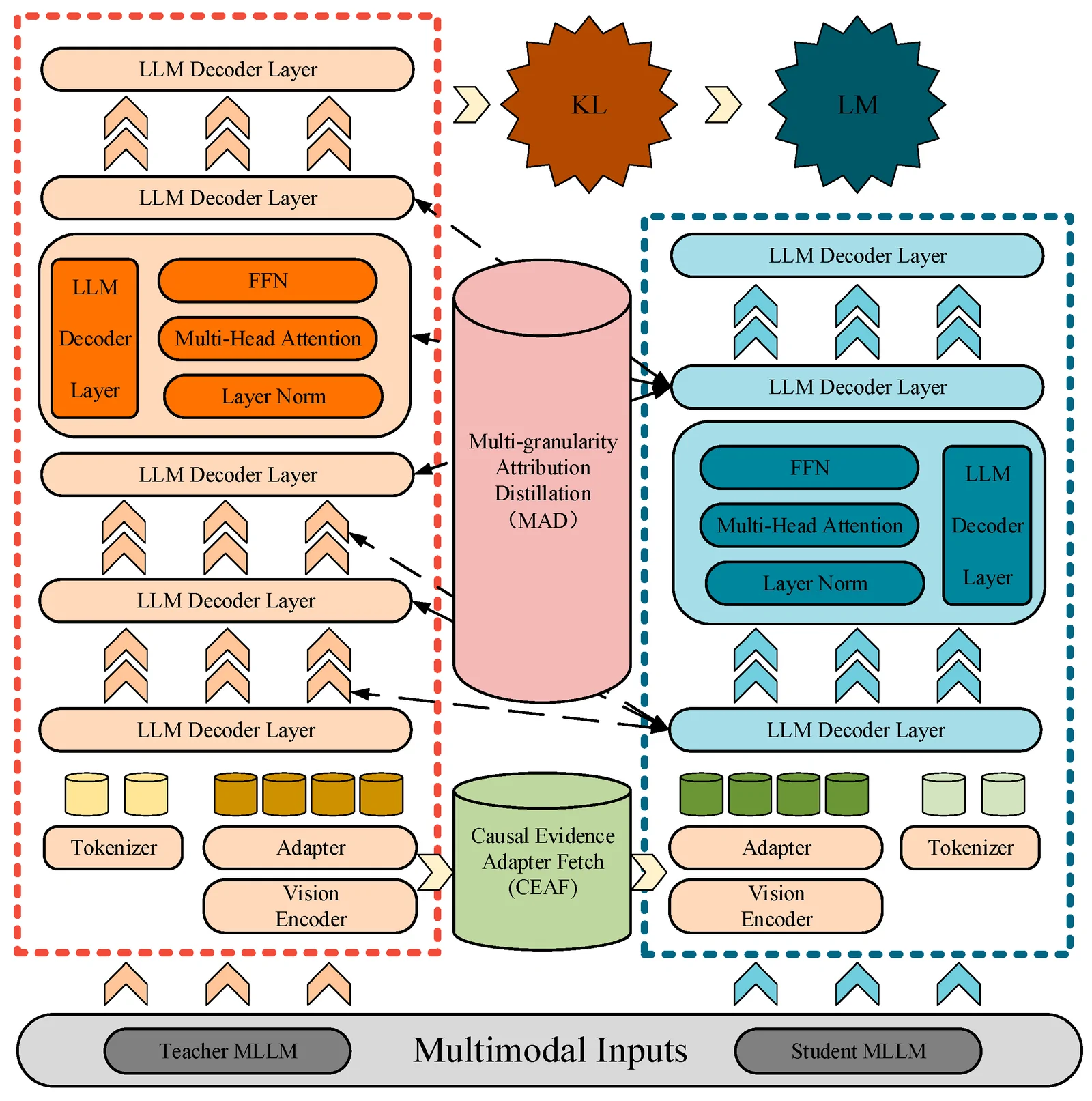
Overall architecture of the proposed CMVAD framework. The framework consists of a teacher MLLM, a student MLLM, the proposed Causal Evidence Adapter Fetch (CEAF), and the Multi-granularity Attribution Distillation (MAD) module. CEAF extracts decision-critical visual evidence from multiple decoder layers through causal attribution, while MAD transfers the extracted visual evidence to the student model by aligning attribution distributions, salient regions, and evidence ranking across multiple decoder layers. The student model is jointly optimized with language modeling and knowledge distillation objectives.

This section presents the proposed Causal and Multi-source Visual Attribution Distillation framework, as shown in Figure 2. Unlike conventional multimodal knowledge distillation methods, which primarily align output logits or intermediate feature representations, the proposed framework explicitly models the visual evidence selection mechanism employed by multimodal large language models during answer generation. The primary objective is to transfer the decision-critical visual evidence acquired by the teacher model to the student model. Specifically, given an input image *I* and a textual instruction *x*, the teacher model first extracts cross-modal interactions between textual tokens and visual tokens from the decoder’s cross-modal interaction layers and subsequently constructs token-level causal attribution maps through region-level interventions. Based on these causal attributions, a Causal Evidence Adapter Fetch (CEAF) module is introduced to replace conventional attention-correlation-based feature fusion with a causal-evidence-guided aggregation strategy. Finally, the student model predicts the corresponding visual evidence distribution and learns the teacher model’s evidence selection behavior under the joint supervision of attribution distribution distillation, key-region distillation, and ranking distillation (MAD).

The central idea of the proposed framework is that the distillation objective should not be restricted to the teacher model’s final predictions but should additionally capture the visual evidence genuinely responsible for producing those predictions. Accordingly, the proposed method extends the conventional output-alignment paradigm to evidence-oriented alignment.

Let the teacher and student multimodal large language models be denoted by *T* and *S*, respectively. For an input image *I* and a textual instruction *x*, the generated output sequence is formulated as:

(1) *y* = (*y*_1_,*y*_2_,…,*y_M_*),

where *M* denotes the sequence length. Conventional knowledge distillation transfers knowledge by minimizing the discrepancy between the output probability distributions of the teacher and student models at each decoding step:

(2) $$L_{\mathrm{KD}}=\sum_{m=1}^{M}\mathrm{KL}\left(P_{T}\left(y_{m}|I,x,y_{1:m-1}\right)\;∥\;P_{S}\left(y_{m}|I,x,y_{1:m-1}\right)\right).$$

However, this objective constrains only the output probability distributions and therefore cannot guarantee consistency between the teacher and student models with respect to visual region selection, visual evidence utilization, and cross-modal reasoning. To address this limitation, visual attribution supervision is further incorporated, yielding the following unified optimization objective:

(3) ℒ = ℒ*_KD_* + *λ_a_*ℒ*_attr_*,

where ℒ*_attr_* denotes the attribution distillation loss and *λ_a_* represents the corresponding balancing coefficient. Compared with conventional knowledge distillation methods, the key distinction of the proposed framework is that ℒ*_attr_* does not simply enforce the similarity of attention maps. Instead, it explicitly models and transfers the teacher model’s causal visual evidence distribution, enabling the student model to acquire a more faithful and interpretable visual reasoning process.

### 3.1. Token-Level Visual Evidence Construction via Region Interventions

This section presents the construction of token-level causal visual attributions from the teacher model. Unlike conventional approaches that directly employ attention maps as visual supervision, the proposed method estimates the actual contribution of each visual region to the model prediction through region-level interventions. The underlying intuition is straightforward: if a visual region is essential for predicting a specific textual token, masking or replacing that region should lead to a substantial reduction in the model’s confidence for that token.

The input image *I* is first partitioned into *N* visual regions:

(4) *I* = {*r*_1_,*r*_2_,…,*r_N_*}.

Each region *r_j_* may correspond either to a fixed-size image patch or to a semantic region generated by a region proposal algorithm. For every region *r_j_*, an intervened image *I*^−^*^j^* is constructed as:

(5) *I*^−^*^j^* = ℳ(*I*,*r_j_*),

where ℳ(⋅) denotes the region intervention operator, which may be implemented through masking, blurring, replacement with the mean pixel value, or substitution using a background patch. The proposed framework focuses on the prediction variation caused by removing the visual information rather than on the intervention strategy itself. Therefore, any intervention capable of effectively suppressing the visual content of region *r_j_* can be employed to estimate its causal contribution.

For a textual token *t_i_*, let the prediction score produced by the teacher model under the complete input (*I*,*x*) be defined as:

(6) $$s_{i}^{T}\left(I,x\right)=\log P_{T}\left(t_{i}|I,x,t_{1:i-1}\right),$$

(7) $$s_{i}^{T}\left(I^{-j},x\right)=\log P_{T}\left(t_{i}|I^{-j},x,t_{1:i-1}\right).$$

Accordingly, the causal contribution of region *r_j_* to token *t_i_* is computed as:

(8) $$A_{i}^{\mathrm{causal}}\left(r_{j}\right)=s_{i}^{T}\left(I,x\right)-s_{i}^{T}\left(I^{-j},x\right).$$

A larger value of $A_{i}^{\mathrm{causal}}\left(r_{j}\right)$ indicates that masking region *r_j_* significantly decreases the prediction confidence of token *t_i_*, implying that this region provides strong causal evidence for generating the corresponding token *t_i_*. Conversely, if $A_{i}^{\mathrm{causal}}\left(r_{j}\right)$ is close to zero, the associated visual region contributes only marginally to the prediction.

(9) $${\hat{A}}_{i}^{\mathrm{causal}}\left(r_{j}\right)=\max\left(A_{i}^{\mathrm{causal}}\left(r_{j}\right),0\right).$$

To alleviate the influence of negative values and extreme outliers during subsequent distillation, the original attribution scores are transformed into a non-negative representation:

(10) $${\tilde{A}}_{i}^{\mathrm{causal}}\left(r_{j}\right)=\frac{{\hat{A}}_{i}^{\mathrm{causal}}\left(r_{j}\right)}{\sum_{k=1}^{N}{\hat{A}}_{i}^{\mathrm{causal}}\left(r_{k}\right)+\epsilon },$$

where *ϵ* is a small constant introduced to prevent numerical instability caused by a zero denominator. Furthermore, a temperature coefficient *τ* is incorporated to regulate the smoothness of the attribution distribution:

(11) $$P_{i}^{\mathrm{causal}}\left(r_{j}\right)=\frac{\exp\left(\frac{{\tilde{A}}_{i}^{\mathrm{causal}}\left(r_{j}\right)}{\tau }\right)}{\sum_{k=1}^{N}\exp\left(\frac{{\tilde{A}}_{i}^{\mathrm{causal}}\left(r_{k}\right)}{\tau }\right)}.$$

A smaller value of *τ* produces a sharper distribution that emphasizes a few highly informative regions, whereas a larger value yields a smoother distribution that better captures the overall evidence structure. After the above processing, each textual token is associated with a probabilistic causal visual evidence distribution:

(12) $$P_{i}^{\mathrm{causal}}=\left[P_{i}^{\mathrm{causal}}\left(r_{1}\right),P_{i}^{\mathrm{causal}}\left(r_{2}\right),\ldots ,P_{i}^{\mathrm{causal}}\left(r_{N}\right)\right].$$

Finally, the complete set of token-level causal visual attributions generated by the teacher model is represented as:

(13) $$P^{\mathrm{causal}}=\left\{P_{1}^{\mathrm{causal}},P_{2}^{\mathrm{causal}},\ldots ,P_{L}^{\mathrm{causal}}\right\}.$$

Compared with conventional attention maps, token-level causal attribution provides substantially stronger interpretability for model decisions. While attention maps merely characterize the correlation between textual and visual tokens during feature interaction, causal attribution explicitly answers whether removing a visual region changes the model prediction. Consequently, the proposed framework adopts token-level causal attribution as the fundamental supervisory signal for subsequent feature fusion and knowledge distillation.

### 3.2. Causal Evidence Adapter Fetch (CEAF)

**Figure 3:**
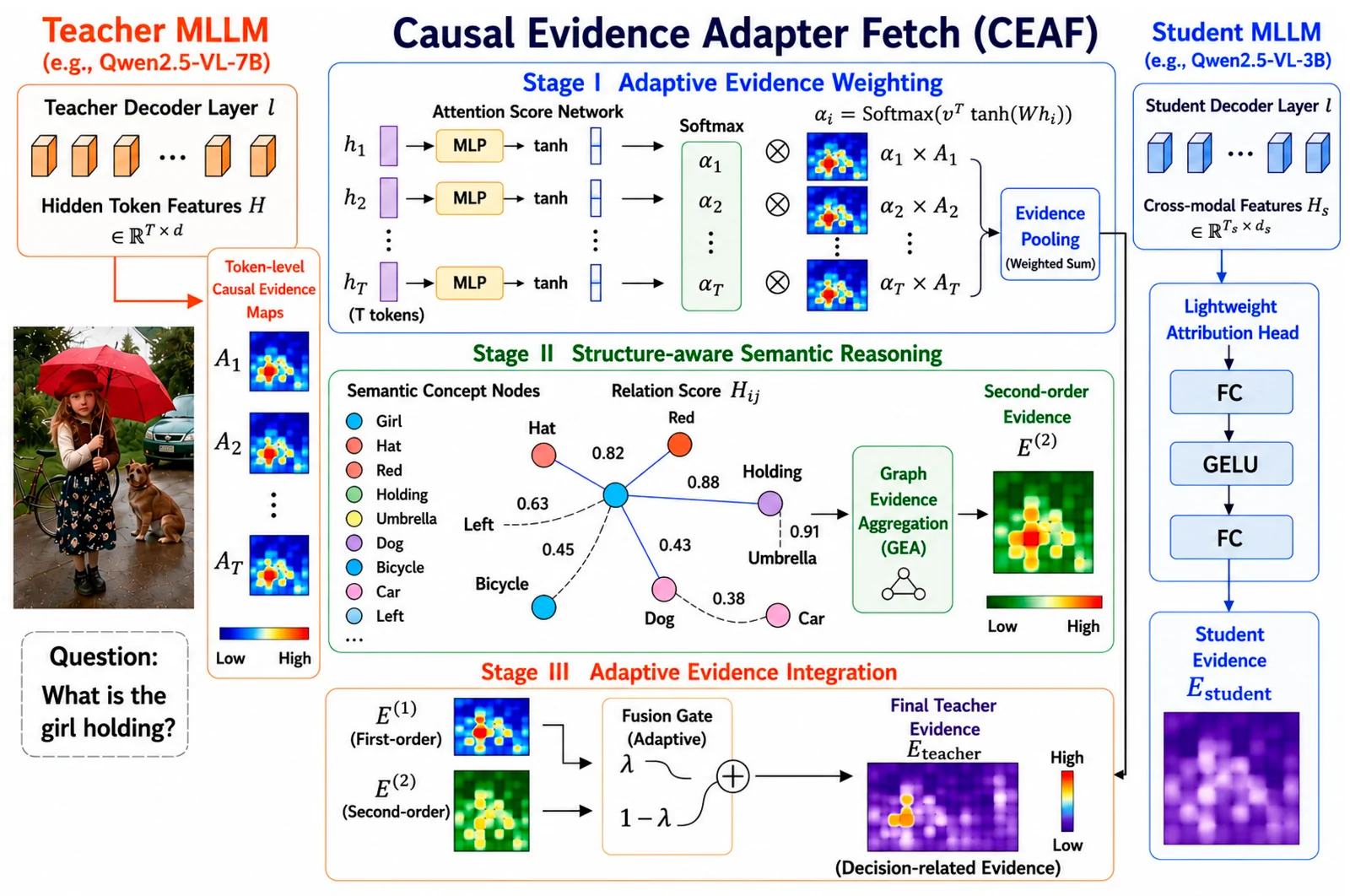
Overall architecture of the proposed CEAF framework. CEAF extracts decision-related visual evidence from the teacher MLLM through adaptive evidence weighting, semantic reasoning, and evidence integration, and supervises the student model using the proposed MAD objectives for visual evidence distillation.

This section introduces the proposed Causal Evidence Adapter Fetch (CEAF) module, as shown in Figure 3. The central idea of CEAF is to replace the direct fusion of token-level attention maps with token-level causal evidence maps, thereby upgrading the fusion target from superficial attention patterns to decision-relevant visual evidence.

Given the token-level causal evidence distributions obtained in the previous section:

(14) $$P^{\mathrm{causal}}=\left\{P_{1}^{\mathrm{causal}},P_{2}^{\mathrm{causal}},\ldots ,P_{L}^{\mathrm{causal}}\right\},$$

An adaptive fusion weight is first computed for each textual token. Let $h_{i}^{T}$ denote the hidden representation of the *i*-th textual token in the teacher model. The corresponding token importance score is defined as:

(15) $$e_{i}=q^{⊤}\sigma \left(W_{h}h_{i}^{T}+b_{h}\right),$$

where *W_h_* and *b_h_* denote learnable parameters, *q* represents the projection vector, and *σ*(⋅) is a nonlinear activation function. The normalized token weights are then obtained through the Softmax function:

(16) $$w_{i}=\frac{\exp\left(e_{i}\right)}{\sum_{k=1}^{L}\exp\left(e_{k}\right)}.$$

Based on these token weights, the causal evidence distributions associated with different tokens are aggregated as follows:

(17) $$P_{\mathrm{fusion}}^{\mathrm{causal}}=\sum_{i=1}^{L}w_{i}P_{i}^{\mathrm{causal}}.$$

This formulation allows semantically important tokens to contribute more significantly to the final visual evidence distribution. For example, in the question “What is the person wearing a red hat holding?”, tokens such as “red hat” and “holding” play a dominant role in object localization and recognition and should therefore receive higher fusion weights.

Nevertheless, first-order token weighting alone is insufficient for modeling the compositional semantics required by complex multimodal reasoning. Numerous vision-language tasks depend on phrase-level or relational semantics, such as “red cup,” “the person on the left,” or “the girl holding an umbrella.” Such semantic concepts emerge from interactions among multiple tokens rather than individual tokens. To address this issue, a structure-aware token interaction mechanism is further introduced. For any pair of textual tokens *t_i_* and *t_j_*, the interaction weight is defined as:

(18) $$w_{\mathrm{ij}}=\frac{\exp\left(\phi \left(h_{i}^{T},h_{j}^{T}\right)\right)}{\sum_{p=1}^{L}\sum_{q=1}^{L}\exp\left(\phi \left(h_{p}^{T},h_{q}^{T}\right)\right)},$$

where *ϕ*(⋅,⋅) denotes the pairwise relation scoring function, which can be implemented using a bilinear formulation:

(19) $$\phi \left(h_{i}^{T},h_{j}^{T}\right)={\left(h_{i}^{T}\right)}^{⊤}W_{r}h_{j}^{T}.$$

The corresponding pairwise causal evidence representation is formulated as:

(20) $$P_{\mathrm{ij}}^{\mathrm{pair}}=\psi \left(P_{i}^{\mathrm{causal}},P_{j}^{\mathrm{causal}}\right),$$

where *ψ*(⋅) denotes the evidence fusion operator. In this work, element-wise multiplication is adopted to emphasize the visual regions jointly attended by the two interacting tokens:

(21) $$P_{\mathrm{ij}}^{\mathrm{pair}}=\frac{P_{i}^{\mathrm{causal}}⊙P_{j}^{\mathrm{causal}}}{\sum_{k=1}^{N}\left(P_{i}^{\mathrm{causal}}\left(r_{k}\right)P_{j}^{\mathrm{causal}}\left(r_{k}\right)\right)+\epsilon }.$$

Accordingly, the structured token interaction evidence is represented as:

(22) $$P_{\mathrm{struct}}^{\mathrm{causal}}=\sum_{i=1}^{L}\sum_{j=1}^{L}w_{\mathrm{ij}}P_{\mathrm{ij}}^{\mathrm{pair}}.$$

The final teacher-side visual evidence distribution is obtained by combining the first-order token evidence and the second-order structured evidence:

(23) $$P_{T}^{\mathrm{evi}}=\eta P_{\mathrm{fusion}}^{\mathrm{causal}}+\left(1-\eta \right)P_{\mathrm{struct}}^{\mathrm{causal}},$$

where *η* ∈ [0, 1] controls the trade-off between first-order token evidence and structured token interaction evidence. To ensure that $P_{T}^{\mathrm{evi}}$ satisfies the properties of a probability distribution during knowledge distillation, it is normalized once again:

(24) $$P_{T}^{\mathrm{evi}}\left(r_{j}\right)=\frac{P_{T}^{\mathrm{evi}}\left(r_{j}\right)}{\sum_{k=1}^{N}P_{T}^{\mathrm{evi}}\left(r_{k}\right)+\epsilon }.$$

Unlike the teacher model, the student model does not perform computationally expensive region-level interventions. Instead, a lightweight attribution prediction head is introduced to directly estimate the visual evidence distribution from the cross-modal decoder representations. Let $Z_{l}^{S}$ denote the cross-attention output of the *l*-th layer in the student model. The student-side evidence distribution is formulated as:

(25) $$P_{S}^{\mathrm{evi}}=\mathrm{softmax}\left(g_{\theta }\left(Z_{l}^{S}\right)\right),$$

where *g_θ_*(⋅) denotes the attribution prediction head of the student model. This design eliminates repeated region interventions during student training, thereby substantially reducing computational overhead. Given the teacher evidence distribution $P_{T}^{\mathrm{evi}}$ and the student evidence distribution $P_{S}^{\mathrm{evi}}$, global attribution distribution distillation is first performed using the Kullback–Leibler divergence:

(26) $$L_{\mathrm{dist}}=\mathrm{KL}\left(P_{T}^{\mathrm{evi}}\;∥\;P_{S}^{\mathrm{evi}}\right)=\sum_{j=1}^{N}P_{T}^{\mathrm{evi}}\left(r_{j}\right)\log\frac{P_{T}^{\mathrm{evi}}\left(r_{j}\right)}{P_{S}^{\mathrm{evi}}\left(r_{j}\right)+\epsilon }.$$

Furthermore, to encourage the student model to focus on the most informative visual regions, the top-*K* regions with the highest confidence scores in the teacher evidence distribution are selected:

(27) $$\Omega_{T}=\mathrm{TopK}\left(P_{T}^{\mathrm{evi}},K\right).$$

The key-region distillation loss is defined as:

(28) $$L_{\mathrm{topk}}=-\frac{1}{K}\sum_{r_{j}\in \Omega_{T}}\log P_{S}^{\mathrm{evi}}\left(r_{j}\right).$$

Compared with the global distribution constraint imposed by ℒ*_dist_*, the loss ℒ*_topk_* explicitly emphasizes local decision-critical visual evidence.

Finally, a ranking distillation objective is introduced to preserve the relative importance among visual regions. For any pair of regions (*r_i_*,*r_j_*), if the teacher model considers *r_i_* to be more important than *r_j_*, namely $P_{T}^{\mathrm{evi}}\left(r_{i}\right)>P_{T}^{\mathrm{evi}}\left(r_{j}\right)$, the student model is encouraged to maintain the same ordering. The set of region pairs is defined as:

(29) $$R=\left\{\left(i,j\right)|P_{T}^{\mathrm{evi}}\left(r_{i}\right)>P_{T}^{\mathrm{evi}}\left(r_{j}\right)\right\}.$$

The ranking distillation loss is formulated as:

(30) $$L_{\mathrm{rank}}=\frac{1}{\left|R\right|}\sum_{\left(i,j\right)\in R}\max\left(0,\delta -\left(P_{S}^{\mathrm{evi}}\left(r_{i}\right)-P_{S}^{\mathrm{evi}}\left(r_{j}\right)\right)\right),$$

where *δ* denotes the ranking margin. This objective enables the student model not only to identify important visual regions but also to learn their relative importance.

Overall, the proposed CEAF module offers two major advantages. First, it transfers causally meaningful visual evidence instead of directly distilling unreliable attention patterns. Second, by jointly modeling token-level weighting and pairwise token interactions, it effectively captures compositional semantics, making the proposed framework more suitable for complex multimodal reasoning tasks.

### 3.3. Multi-Granularity Attribution Distillation (MAD)

**Figure 4:**
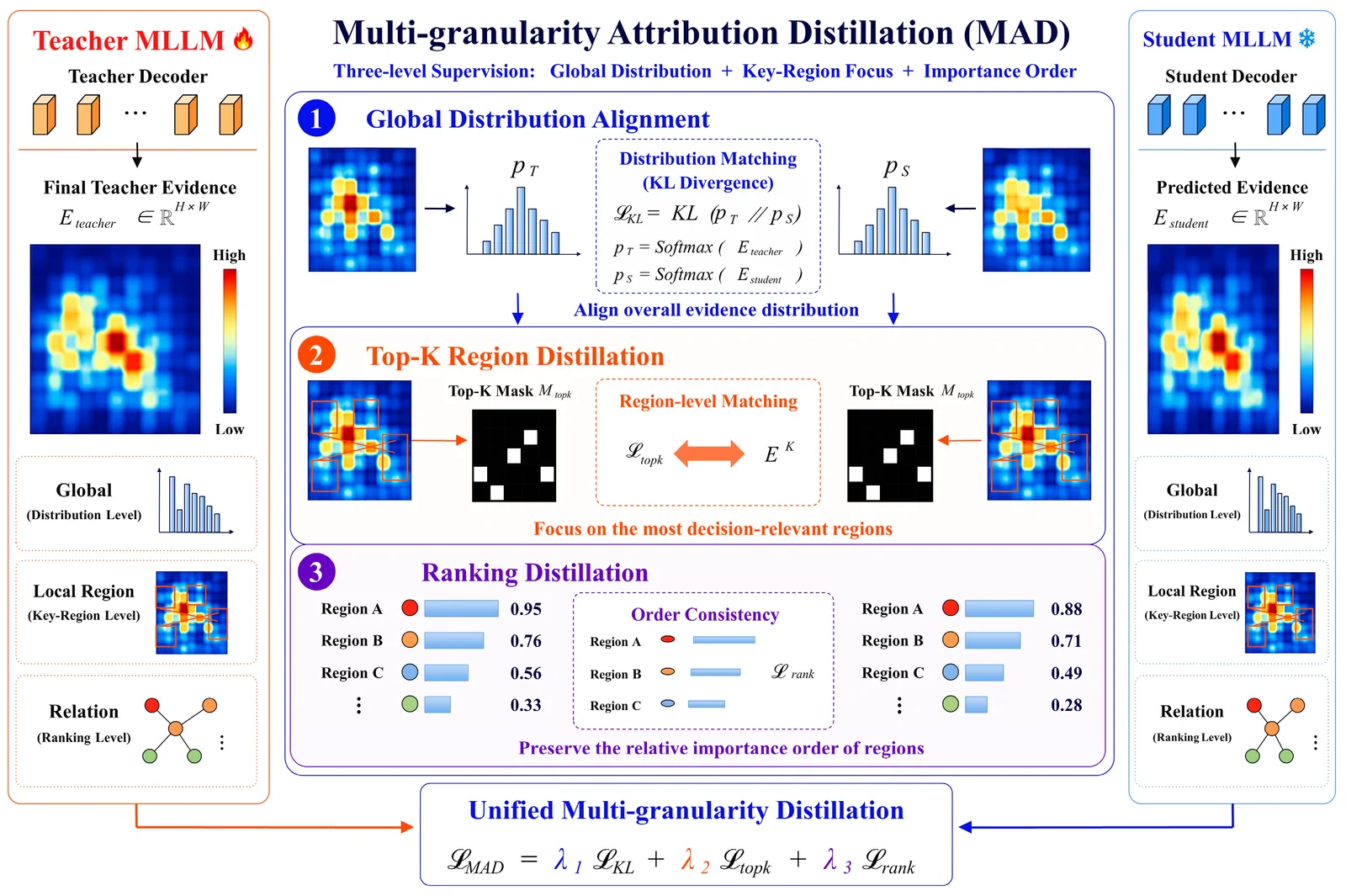
Illustration of the proposed Multi-granularity Attribution Distillation (MAD) framework. MAD performs visual evidence transfer by jointly aligning global evidence distributions, decision-critical regions, and region importance rankings, thereby enhancing the visual perception and reasoning capabilities of the student MLLM.

The causal-aware token attribution fusion module produces the final visual evidence distribution of the teacher model. Unlike a conventional attention map, this representation integrates token-level causal attribution, token-importance weighting, and structured token interactions, thereby providing decision-oriented visual evidence. To effectively transfer this knowledge to the student model, we propose the Multi-granularity Attribution Distillation (MAD) framework, as shown in Figure 4.

Employing only a single mean squared error or Kullback–Leibler (KL) divergence to align teacher and student attribution maps generally enables the student model to capture only a coarse approximation of the global distribution, while failing to reliably identify the truly discriminative visual regions exploited by the teacher. In multimodal compositional reasoning, visual evidence naturally exhibits a multi-granularity structure. Specifically, the teacher model forms a global evidence distribution across the entire image, the final prediction is primarily dominated by a small number of critical regions, and the relative importance among different regions also conveys valuable supervisory information. Accordingly, MAD supervises the student model from three complementary perspectives: global distribution, local critical regions, and relative ranking relationships.

The distribution of evidence obtained from the teacher side is:

(31) $$P_{T}^{A}=\mathrm{Norm}\left(A_{T}^{\mathrm{final}}\right)\in R^{N},$$

The corresponding evidence distribution predicted by the student model is formulated as:

(32) $$P_{S}^{A}=g_{S}\left(H_{S},V_{S}\right)\in R^{N},$$

where *H_S_* and *V_S_* denote the textual and visual representations of the student model, respectively, and *g_S_*(⋅) represents the lightweight attribution prediction head. The objective of MAD is to encourage the student model not only to approximate the overall evidence distribution of the teacher but also to accurately identify the most informative decision regions while preserving the relative importance among visual regions.

The overall optimization objective is defined as:

(33) ℒ*_MAD_* = ℒ*_kl_* + *α*ℒ*_topk_* + *β*ℒ*_rank_*,

where ℒ*_kl_*, ℒ*_topk_*, and ℒ*_rank_* denote the attribution distribution distillation loss, key-region distillation loss, and ranking-consistency distillation loss, respectively, while *α* and *β* are balancing coefficients.

#### 3.3.1. Global Attribution Distribution Distillation

Global attribution distribution distillation enforces consistency between the teacher and student in terms of the overall visual evidence distribution. Since both teacher and student outputs represent probability distributions over visual regions, the KL divergence is adopted:

(34) $$L_{\mathrm{kl}}=D_{\mathrm{KL}}\left(P_{T}^{A}\|P_{S}^{A}\right)=\sum_{j=1}^{N}P_{T}^{A}\left(j\right)\log\frac{P_{T}^{A}\left(j\right)}{P_{S}^{A}\left(j\right)+\epsilon },$$

where *ϵ* is a small constant introduced to ensure numerical stability. To further improve optimization stability, temperature smoothing is applied before knowledge distillation. The teacher and student distributions are respectively expressed as:

(35) $${\tilde{P}}_{T}^{A}\left(j\right)=\frac{\exp\left(\frac{A_{T}^{\mathrm{final}}\left(j\right)}{\tau }\right)}{\sum_{k=1}^{N}\exp\left(\frac{A_{T}^{\mathrm{final}}\left(k\right)}{\tau }\right)},$$

(36) $${\tilde{P}}_{S}^{A}\left(j\right)=\frac{\exp\left(\frac{A_{S}\left(j\right)}{\tau }\right)}{\sum_{k=1}^{N}\exp\left(\frac{A_{S}\left(k\right)}{\tau }\right)},$$

where *τ* denotes the temperature coefficient. A smaller temperature produces a sharper distribution, emphasizing highly informative regions, whereas a larger temperature yields a smoother distribution that facilitates learning of the complete evidence structure. The temperature-aware distribution distillation objective is therefore given by:

(37) $$L_{\mathrm{kl}}^{\tau }=\tau^{2}D_{\mathrm{KL}}\left({\tilde{P}}_{T}^{A}\|{\tilde{P}}_{S}^{A}\right).$$

The additional scaling factor *τ*^2^ preserves gradient magnitude during optimization. Although this objective effectively aligns the global evidence distribution, it cannot explicitly guarantee that the student focuses on the most critical visual regions.

#### 3.3.2. Key-Region Distillation

In multimodal reasoning, final predictions are typically dominated by only a few critical visual regions. Therefore, key-region distillation is introduced to explicitly strengthen the student’s attention to the core decision regions identified by the teacher.

The top-*K* regions with the highest teacher evidence scores are first selected:

(38) $$\Omega_{T}^{K}=\mathrm{TopK}\left(P_{T}^{A},K\right),$$

A binary region mask is subsequently constructed:

(39) $$M_{T}\left(j\right)=\left\{\begin{array}{ll} 1, & j\in \Omega_{T}^{K},\\ 0, & j\notin \Omega_{T}^{K}. \end{array}\right.$$

The key-region distillation loss is defined as:

(40) $$L_{\mathrm{topk}}=-\frac{1}{K}\sum_{j\in \Omega_{T}^{K}}\log\left(P_{S}^{A}\left(j\right)+\epsilon \right).$$

Unlike global distribution alignment, this objective explicitly encourages the student model to allocate higher confidence to the most informative regions identified by the teacher.

To suppress excessive responses on irrelevant regions, an additional background suppression term is incorporated:

(41) $$L_{\mathrm{neg}}=-\frac{1}{N-K}\sum_{j\notin \Omega_{T}^{K}}\log\left(1-P_{S}^{A}\left(j\right)+\epsilon \right).$$

The final key-region distillation objective is expressed as:

(42) $$L_{\mathrm{topk}}^{*}=L_{\mathrm{topk}}+\eta L_{\mathrm{neg}},$$

#### 3.3.3. Ranking-Consistency Distillation

While global distribution and key-region distillation constrain the overall and local evidence representations, neither explicitly models the relative importance among visual regions. To preserve this structural information, ranking-consistency distillation is proposed.

A set of informative region pairs is first constructed according to the teacher evidence distribution:

(43) $$R_{T}=\left\{\left(i,j\right)|P_{T}^{A}\left(i\right)>P_{T}^{A}\left(j\right)+\delta \right\},$$

where *δ* denotes the ranking margin threshold used to discard ambiguous region pairs.

For every valid region pair, the student model is expected to preserve the same ranking relationship as the teacher. The ranking distillation loss is defined as:

(44) $$L_{\mathrm{rank}}=\frac{1}{\left|R_{T}\right|}\sum_{\left(i,j\right)\in R_{T}}\max\left(0,m-P_{S}^{A}\left(i\right)+P_{S}^{A}\left(j\right)\right),$$

where *m* represents the ranking margin. This objective penalizes inconsistent ranking relationships and encourages the student model to preserve the relative importance among visual regions.

Overall, MAD transfers the teacher’s visual evidence through three complementary supervisory granularities. Global distribution distillation captures the holistic evidence structure, key-region distillation emphasizes the most discriminative decision regions, and ranking-consistency distillation preserves the relative importance among regions. Consequently, MAD should be viewed as a unified multi-granularity evidence transfer framework rather than a simple combination of multiple loss functions.

## 4. Experiments and Analysis

### 4.1. Experimental Settings

The proposed framework follows a three-stage training pipeline consisting of distilled pre-training (DPT), supervised fine-tuning (SFT), and distilled fine-tuning (DFT). Visual inputs are encoded by the SigLIP-so400m-patch14-384 encoder and projected into the language-model embedding space through a two-layer multilayer perceptron (MLP) with GELU activation. The vision encoder remains frozen throughout all three training stages. The DPT stage uses the LLaVA-Pretrain-558K dataset with a batch size of 32 and an initial learning rate of 1 × 10^−3^. The SFT and DFT stages both use the LLaVA-v1.5-Mix-665K dataset with a batch size of 16 and an initial learning rate of 2 × 10^−5^. Each stage is trained for one epoch using the AdamW optimizer and a cosine-decay learning-rate schedule.

The DPT and DFT stages jointly optimize the standard autoregressive language-modeling loss, response-token distribution distillation loss, visual-token distribution distillation loss, and visual-token relational distillation loss. The corresponding loss weights are set to α = α′ = 1.0, β = β′ = 1.0, and γ = γ′ = 0.5. The experimental environment is based on Python 3.12, PyTorch 2.6.0, and CUDA 12.6.

To transfer text-generation capability, response-token distributions, visual-token distributions, and relational information among visual tokens, the DPT stage employs a joint objective consisting of four loss components. The total DPT loss is defined as

(45) *L_DPT_* = *L_reg_* + *αL_res_* + *βL_vis_* + *γL_rel_*

The DFT stage uses the same loss components while adopting independent weighting coefficients. Its total loss is defined as

(46) *L_DFT_* = *L_reg_* + *α*′*L_res_* + *β*′*L_vis_* + *γ*′*L_rel_*

Region-level interventions are performed exclusively during training. For each input sample, the teacher model processes the original image together with multiple intervened images generated by masking or modifying specified visual regions. When K intervention regions are used and token-specific causal evidence is estimated for T generated tokens, the teacher performs one original-image evaluation and K additional intervention evaluations for each generated token. Accordingly, the total number of teacher evaluations is represented as *T*(*K* + 1).

The complete training pipeline includes student-parameter optimization, teacher-side intervention inference, evidence extraction, and distillation-loss computation and requires approximately 240 GPU-hours in total. Because the intervention procedure is integrated into the training pipeline rather than executed as an independent preprocessing stage, its computational cost is included in the overall training budget and is not reported as a separate preprocessing time.

Student-model inference is evaluated separately from the training process. Before deployment, the teacher model, region-intervention module, cached causal evidence, and auxiliary attribution head are removed. The deployed student model performs inference solely through its standard vision-language forward pass. Under the inference configuration used in this work, the student model requires approximately 7.9 GB of peak GPU memory, and the computational cost of a single inference is approximately 2.9 TFLOPs. These measurements indicate that the proposed framework introduces additional computational overhead during training, whereas the auxiliary attribution components do not increase the deployment-time inference cost of the student model.

### 4.2. Experimental Dataset

To comprehensively evaluate both the visual perception capability and compositional reasoning ability of the proposed method, the experimental benchmarks are divided into two categories: General Visual Question Answering (VQA) and Compositional Reasoning (CR) tasks. General VQA benchmarks primarily assess whether a model can accurately recognize objects, scenes, attributes, and textual content within images, thereby reflecting its fundamental visual understanding capability. In contrast, CR benchmarks place greater emphasis on high-level multimodal reasoning by evaluating the model’s ability to understand object relationships, attribute binding, spatial reasoning, and complex semantic compositions. Consequently, these two categories provide complementary evaluations of low-level visual perception and high-level reasoning performance.

For the General VQA evaluation, three widely adopted public benchmarks, namely GQA [8], TextVQA [26], and MME [27], are employed. Specifically, GQA evaluates structured scene understanding and relational reasoning by requiring models to infer object interactions and compositional semantics. TextVQA measures the capability of multimodal models to recognize and reason over textual information embedded in natural images, thereby emphasizing optical character recognition and language–vision integration. MME provides a comprehensive evaluation framework covering both perceptual and cognitive abilities of multimodal large language models, making it a widely adopted benchmark for evaluating the general multimodal intelligence of modern MLLMs.

For the Compositional Reasoning (CR) task, experiments are conducted on the SugarCrepe benchmark [9], which is specifically designed to evaluate fine-grained visual compositional understanding. Unlike conventional VQA datasets, SugarCrepe requires models to distinguish subtle semantic differences arising from object attributes, spatial relationships, and compositional structures. Therefore, it serves as an effective benchmark for assessing whether the proposed causal evidence distillation framework can enhance the compositional reasoning capability of the student model.

### 4.3. Comparison Experiments

**Figure 5:**
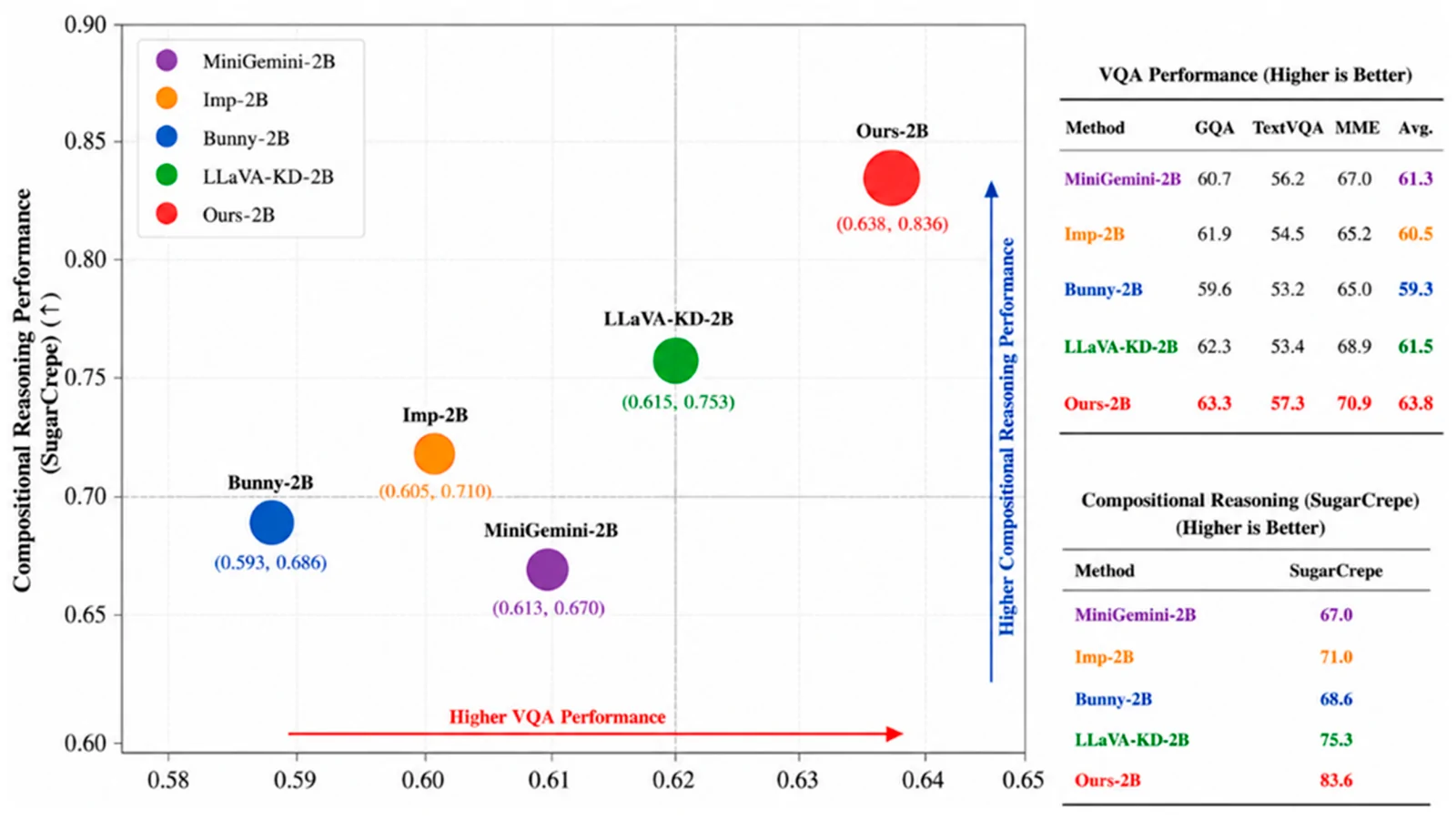
Comparison of KD methods.

As summarized in Table 1, we comprehensively compare the proposed CMVAD with representative multimodal large language models (MLLMs) of different parameter scales on both General Visual Question Answering (VQA) and Compositional Reasoning (CR) benchmarks. Despite maintaining a relatively small model size (Qwen1.5-1.8B, approximately 2B parameters), the proposed method achieves highly competitive overall performance, demonstrating the effectiveness of the proposed causal-aware visual attribution distillation framework, as shown in Figure 5.

On the GQA benchmark, CMVAD achieves an accuracy of 63.3%, outperforming LLaVA-KD-2B by 1.0 percentage point, while surpassing Imp-2B by 1.4 percentage points, respectively. Compared with Bunny-2B, the performance gain further increases to 3.7 percentage points. Since GQA primarily evaluates relational reasoning and structured scene understanding, these improvements indicate that the proposed attribution-driven distillation strategy enables the student model to acquire more discriminative visual relationship representations, thereby enhancing its capability for complex visual semantic reasoning.

On the TextVQA benchmark, the proposed method achieves 57.3%, representing improvements of 3.9 and 2.8 percentage points over LLaVA-KD-2B and Imp-2B, respectively. As TextVQA focuses on scene text recognition and multimodal reasoning, the superior performance suggests that the proposed causal attribution supervision effectively guides the student model to attend to semantically critical textual regions, leading to more accurate modeling of fine-grained visual information.

On the VizWiz benchmark, our model achieves a score of 44.3%, exceeding Imp-2B by 5.1 percentage points, respectively. VizWiz constitutes a particularly demanding evaluation setting, as its images are captured by blind users in unconstrained real-world environments and may contain blur, inadequate illumination, partial occlusion, incomplete objects, or insufficient visual evidence for a determinate answer. These conditions require the model to distinguish informative regions from unreliable visual content while making difficult predictions. The improvement over comparable student models suggests that causal attribution supervision enables the student to retain and exploit localized, decision-relevant visual evidence under noisy and incomplete observation conditions, thereby improving its reliability on challenging visual reasoning cases.

For the comprehensive multimodal evaluation benchmark MME, CMVAD obtains an average score of 70.9%, exceeding LLaVA-KD-2B by 2.0 percentage points and MiniGemini-2B by 3.9 percentage points. Since MME jointly evaluates multiple perception and cognition capabilities, including object perception, optical character recognition (OCR), commonsense reasoning, and multimodal understanding, these results demonstrate that the proposed framework not only improves local visual evidence modeling but also consistently enhances the overall visual perception capability of compact MLLMs.

Overall, CMVAD achieves the highest average performance of 63.8%, outperforming LLaVA-KD-2B (61.5%) and MiniGemini-2B (61.3%) by 2.3 and 2.5 percentage points, respectively. Notably, these improvements are achieved without increasing the model size, highlighting the superior efficiency of the proposed distillation framework. The consistent performance gains across multiple benchmarks demonstrate that causal-aware visual attribution distillation effectively alleviates the insufficient transfer of visual knowledge commonly observed in conventional knowledge distillation methods, enabling the student model to learn more discriminative and semantically meaningful visual representations. These results further demonstrate that explicitly transferring causal visual evidence is more effective than relying solely on output alignment or intermediate feature matching for knowledge distillation.

Table 2 further compares the performance of different multimodal large language models on the compositional reasoning benchmark SugarCrepe. As shown in the table, the proposed CMVAD achieves an accuracy of 83.6%, representing the best performance among all MLLMs with approximately 2B parameters.

More specifically, CMVAD outperforms LLaVA-KD-2B by 8.3%, Imp-2B by 12.6%, MiniGemini-2B by 16.6%, and Bunny-2B by 15.0%, respectively. These substantial improvements consistently demonstrate the effectiveness of the proposed causal-aware visual attribution distillation framework in enhancing compositional reasoning.

Compared with the performance gains observed on the general VQA benchmarks, the proposed method exhibits a significantly larger advantage on SugarCrepe. This observation indicates that the proposed framework not only improves the visual perception capability of the student model but, more importantly, substantially strengthens its ability to capture object relationships, attribute binding, and contextual dependencies, which are fundamental to complex multimodal reasoning.

### 4.4. Comparison of Different Distillation Methods and Visual Dependence

To evaluate the effectiveness of the proposed evidence-aware distillation framework, we compare CMVAD with five representative baselines, including KD-2B, LLaVADI-2B, MoD-2B, GBIS-2B, and CompoDistill-2B.

KD-2B is a conventional multimodal knowledge distillation baseline that transfers the teacher’s predictive behavior through output-level and intermediate representation alignment.

LLaVADI-2B is a visual-aware distillation baseline that introduces visual or cross-modal information into the teacher–student alignment process.

LLaVA-MoD-2B. LLaVA-MoD-2B is a model-oriented multimodal distillation method designed to obtain a compact student model while preserving the capabilities of a larger teacher model. Its main objective is to improve the efficiency and generalization of the student model.

GBIS-2B. GBIS-2B denotes the Gaussian-blurring-based intervention variant used in our experiments. It introduces region-level visual perturbation by blurring selected image regions and provides a direct baseline for evaluating intervention-based supervision.

CompoDistill-2B. CompoDistill-2B is a compositional knowledge distillation method that explicitly transfers the teacher’s attention patterns to the student model. Its attention-guided distillation objective encourages the student to reproduce the teacher’s visual attention distribution and cross-modal interaction structure, thereby improving the identification of object attributes, spatial relationships, and compositional semantic dependencies.

All methods employ the same Qwen1.5-1.8B student backbone and are evaluated under a unified experimental protocol. The evaluation is conducted on GQA, TextVQA, MME, and SugarCrepe, which jointly measure general visual question answering, text-rich visual understanding, multimodal reasoning, and compositional reasoning ability.

As shown in Table 3, CMVAD achieves the best performance on all four benchmarks. Compared with the strongest competing baseline on each benchmark, CMVAD improves the results by 0.6, 0.9, 0.8, and 0.7 points, respectively. The proposed method performs well in general multimodal understanding and combinatorial reasoning tasks.

### 4.5. Ablation Study

To verify the effectiveness of the proposed Causal Evidence Adapter Fetch (CEAF) module and the Multi-granularity Attribution Distillation (MAD) module, comprehensive ablation studies are conducted by individually enabling each component.

To ensure a fair comparison, the CEAF-only and MAD-only variants use the same student architecture, training data, optimization schedule, and language modeling objective as the full model. The teacher model is frozen throughout training, and all teacher-derived causal evidence maps, region masks, and ranking targets are detached using a stop-gradient operation.

In the CEAF-only variant, the CEAF module is retained and trained to reconstruct the unified teacher evidence representation. Its auxiliary supervision loss is defined as

(47) *L_CEAF-only_* = *L_LM_* + *λ_C_ L_CEAF_*

where *L_LM_* is the standard autoregressive language modeling loss. No top-K region alignment loss or ranking consistency loss is applied in this variant.

In the MAD-only variant, CEAF is removed, and the multi-granularity attribution losses are directly imposed on the student evidence maps. The objective is defined as

(48) *L_MAD-only_* = *L_LM_* + *λ*_1_*L_global_* + *λ*_2_*L_topk_* + *λ*_3_*L_rank_*

The experimental results are summarized in Table 4.

When only the CEAF module is incorporated into the baseline model, consistent performance improvements are observed across all evaluation benchmarks. Specifically, the accuracy on GQA increases from 57.8% to 58.6%, while the performance on TextVQA improves from 50.1% to 52.0%. On the MME benchmark, the score rises from 62.5% to 64.1%. These results indicate that CEAF effectively enhances the reliability of the visual evidence extracted from the teacher model by explicitly modeling causal visual attribution, thereby providing more informative supervision for knowledge transfer.

When only the MAD module is retained, the student model also achieves substantial performance gains over the baseline. Compared with the baseline model, the accuracy improves by 3.2 percentage points on GQA, 5.7 percentage points on TextVQA, 5.5 percentage points on MME. These consistent improvements demonstrate that the proposed multi-granularity attribution distillation strategy effectively transfers the teacher model’s visual knowledge by jointly preserving the global evidence distribution, emphasizing decision-critical regions, and maintaining the relative importance among visual regions.

Overall, the experimental results demonstrate that conventional knowledge distillation based solely on output supervision is insufficient for effectively transferring the visual reasoning capability of multimodal large language models. By integrating causal visual evidence modeling with multi-granularity visual evidence distillation, the proposed framework substantially improves both visual perception and compositional reasoning capabilities of the student model. Therefore, the combination of CEAF and MAD forms a coherent visual attribution-driven knowledge distillation framework, enabling more effective transfer of multimodal knowledge and consistently enhancing the overall performance of compact multimodal large language models.

### 4.6. Scalability Analysis with Different Teacher–Student Configurations

To further evaluate the scalability of the proposed framework under different teacher–student configurations, knowledge distillation experiments are conducted using teacher and student models with varying parameter scales. The quantitative results are summarized in Table 5.

As shown in the table, the overall performance consistently improves as the capacity of the student model increases. When the teacher model is fixed as Qwen1.5-4B, enlarging the student model from Qwen1.5-0.5B to Qwen1.5-1.8B increases the average VQA score from 58.5% to 64.9%, corresponding to an improvement of 6.4 percentage points. Meanwhile, the accuracy on the SugarCrepe benchmark rises from 71.5% to 83.5%, yielding a substantial gain of 12.0 percentage points. These results indicate that a larger student model possesses stronger representation capability and greater model capacity, enabling it to absorb the visual semantic knowledge transferred by the teacher model more effectively. Consequently, significant performance improvements are achieved in both visual question answering and compositional reasoning tasks.

Furthermore, when the student model is fixed at Qwen1.5-1.8B, replacing the teacher model from Qwen1.5-4B with the larger Qwen2.5-7B further improves the distillation performance. Specifically, the average VQA score increases from 64.9% to 65.7%, while the SugarCrepe accuracy improves from 83.5% to 84.5%, corresponding to gains of 0.8 and 1.0 percentage points, respectively. These improvements suggest that a more capable teacher model provides richer visual semantic knowledge and higher-quality supervisory signals, allowing the student model to learn more discriminative visual representations during knowledge distillation.

Overall, the experimental results demonstrate that the final distillation performance is jointly influenced by both the student model capacity and the teacher model capability. Increasing the capacity of the student model serves as the primary factor contributing to performance improvement by enhancing knowledge absorption and representation ability. Meanwhile, employing a stronger teacher model further refines the knowledge transfer process by providing more informative and reliable supervisory signals. These findings verify that the proposed framework generalizes well across different teacher–student configurations and can effectively exploit the rich visual semantic knowledge provided by high-capacity teacher models. Consequently, the proposed method exhibits excellent scalability and strong generalization ability, making it suitable for knowledge distillation across multimodal large language models of different parameter scales.

### 4.7. Visualization Analysis

**Figure 6:**
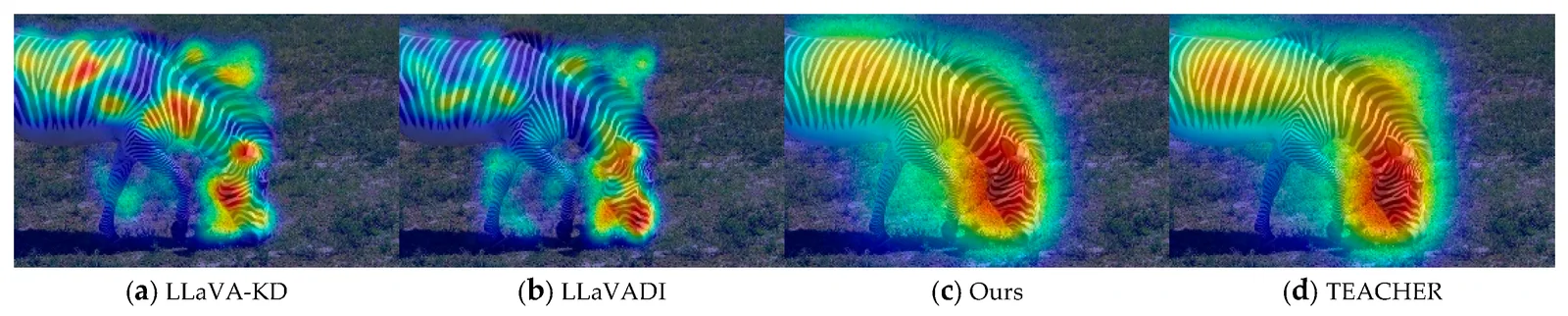
Heatmap visualization for the textual prompt “A zebra is eating grass.” (Warmer colors indicate higher response intensities, whereas cooler colors indicate lower response intensities).

**Figure 7:**
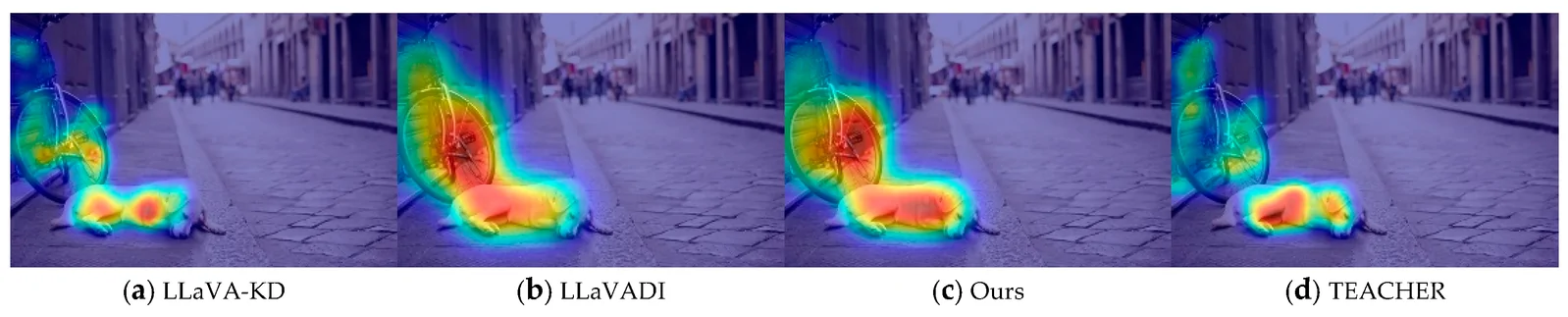
Comparison of heatmap visualizations for the textual prompt “A cat is leaning against a bicycle.”.

To further investigate the visual reasoning behavior of the proposed framework, qualitative comparisons are conducted by visualizing the heatmaps generated by different models. As illustrated in Figure 6 and Figure 7, the heatmaps provide an intuitive representation of the visual evidence utilized during multimodal reasoning, where warmer colors indicate regions receiving stronger responses, while cooler colors correspond to less informative areas.

### 4.8. UAV Object Detection Evaluation

To further evaluate the applicability of the proposed framework to UAV sensor-image object detection, we conduct experiments on the official VisDrone2019-DET benchmark, the results are shown in Table 6. The dataset contains 6471 training images, 548 validation images, and 1610 test-dev images. The training split is used for model optimization, the validation split is used for checkpoint selection, and the test-dev split is reserved exclusively for final evaluation.

All baseline methods are evaluated using their standard benchmark implementations, officially released checkpoints, and default hyperparameters. No additional architectural modules, loss functions, or method-specific improvements are introduced.

Each model is required to generate predictions in the format [category, confidence, x_min, y_min, x_max, y_max], where the box coordinates are normalized to the range [0, 1]. The normalized coordinates are converted into pixel coordinates according to the original image width and height. The converted coordinates are clipped to the valid image boundaries, and predictions with invalid categories, missing values, or non-positive box areas are discarded.

Define the average precision (AP), visually represented by the area under the PR curve on the image. mAP denotes the average AP calculated across all detected object categories.

(49) $$\mathrm{mAP}=\frac{1}{C}\sum_{K=1}^{C}AP_{K}$$

where C is the total number of categories and k represents the category index. In particular, mAP50:95 represents the multithreshold mAP averaged with ten different IoU thresholds, i.e., 0.5 to 0.95.

To examine the reproducibility of the proposed method, CMVAD was independently trained and evaluated three times using different random seeds while keeping the data splits, optimization settings, and evaluation protocol unchanged. As shown in Table 7, CMVAD achieves (62.00 ± 0.70%) mAP@0.50 and (39.00 ± 0.36%) mAP@[0.50:0.95]. The corresponding 95% confidence intervals are ([60.26, 63.74]) and ([38.10, 39.90]), respectively. The relatively small standard deviations indicate that CMVAD maintains stable performance across the independent runs.

**Figure 8:**
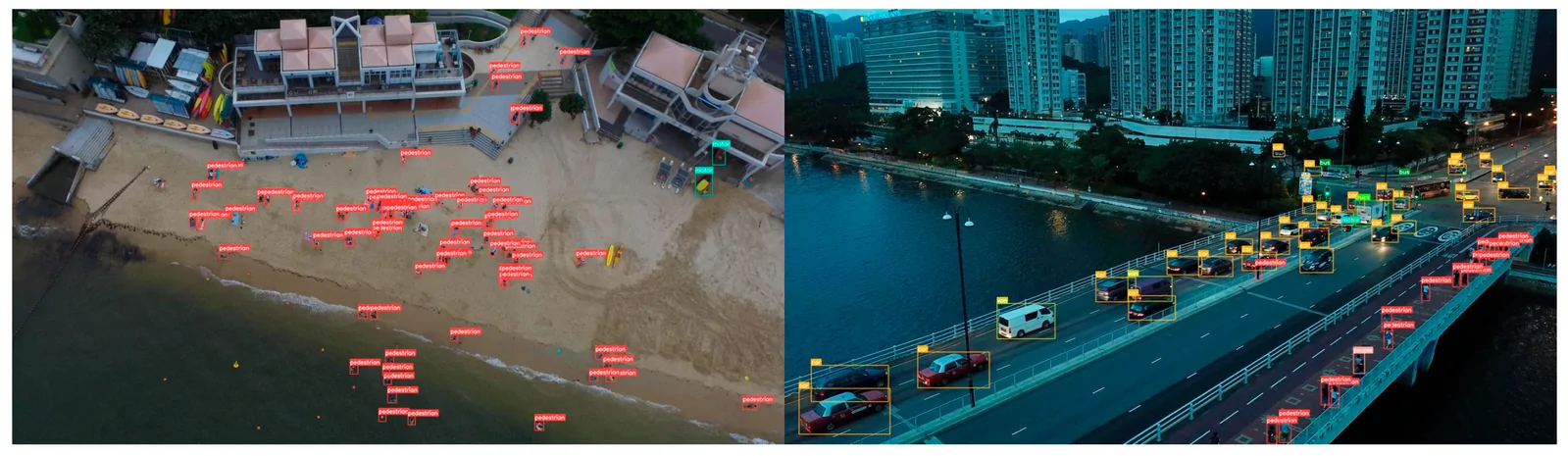
VisDrone dataset detection results.

These findings are consistent with the design of CMVAD, which emphasizes local visual information and its influence on model predictions. By constructing visual supervision through region-level interventions and transferring it through multi-source evidence aggregation and multi-granularity distillation, CMVAD provides supervision complementary to output distribution and intermediate representation alignment. The specific visual detection results are shown in Figure 8.

## 5. Conclusions

This paper presents CMVAD, a causal visual attribution-driven knowledge distillation framework for multimodal large language models. CMVAD constructs token-level visual supervision through region-level interventions, aggregates this supervision using the Causal Evidence Adapter Fetch (CEAF) module, and transfers it to the student through Multi-granularity Attribution Distillation (MAD). By aligning global attribution distributions, salient decision regions, and attribution rankings, the framework supplements conventional output and representation alignment with supervision that reflects the teacher’s dependence on local visual information.

Experiments on public multimodal benchmarks demonstrate the effectiveness of CMVAD. The method achieves an average accuracy of 63.8% on the general VQA benchmarks, exceeding LLaVA-KD by 2.3 percentage points, and obtains 83.6% on SugarCrepe, an improvement of 8.3 percentage points. Ablation studies support the complementary contributions of CEAF and MAD: CEAF improves SugarCrepe accuracy by 5.4 percentage points, while MAD independently yields gains of 5.7 and 5.5 percentage points on TextVQA and MME, respectively. CMVAD also maintains its effectiveness across the evaluated teacher–student configurations. Replacing the Qwen1.5-4B teacher with Qwen2.5-7B further increases SugarCrepe accuracy to 84.5%.

To examine its applicability beyond multimodal understanding, we conduct a supplementary evaluation on the VisDrone UAV object detection benchmark. Together with the detection visualizations, these results extend the empirical assessment of the framework to UAV imagery.

The results demonstrate that intervention-derived visual supervision complements conventional output and representation alignment in multimodal knowledge distillation. By combining token-level visual supervision, multi-source aggregation, and multi-granularity alignment, CMVAD improves student performance on visual question answering and compositional reasoning benchmarks. The supplementary VisDrone evaluation provides further evidence of its applicability to UAV object detection. These findings support the value of explicitly transferring the teacher’s dependence on local visual information to compact multimodal large language models.

## Figures and Tables

**Table 1:** Comparison of multimodal large language models on general visual question answering benchmarks.

Method	LLM	Params	GQA	TextVQA	MME	Avg	VizWiz
LLaVA-1.5	Vicuna-7B	~7B	62.0	58.2	75.5	65.2	-
LLaVA-Next	Vicuna-7B	~7B	64.2	64.9	76.0	68.4	-
MoblieVLM V2	Vicuna-7B	~7B	62.6	62.3	78.0	67.6	-
Qwen2.5-VL-3B	Qwen2-3B	~4B	60.9	68.5	72.8	67.4	-
MobileVLMV2	MLLaMA-2.7B	~4B	61.1	57.5	72.0	63.5	-
Imp-3B	Phi2-2.7B	~4B	63.5	59.8	72.3	65.2	54.1
Tiny LLaVa	Phi2-2.7B	~4B	62.0	59.7	73.2	65.0	-
Bunny-3B	Phi2-2.7B	~4B	62.5	56.7	74.4	64.5	43.8
MoE-LLaVA-3B	Phi2-2.7B	~4B	62.6	57.0	71.6	63.7	43.7
Mini CPM-V	MiniCPM-2.4B	~4B	52.1	73.2	70.5	65.3	60.2
MiniGemini-2B	Gemma-2B	~2B	60.7	56.2	67.0	61.3	41.5
Imp-2B	Qwen1.5-1.8B	~2B	61.9	54.5	65.2	60.5	39.2
Bunny-2B	Qwen1.5-1.8B	~2B	59.6	53.2	65.0	59.3	34.2
LLaVA-KD-2B	Qwen1.5-1.8B	~2B	62.3	53.4	68.9	61.5	44.8
Ours	Qwen1.5-1.8B	~2B	63.3	57.3	70.9	63.8	44.3

**Table 2:** Performance comparison of multimodal large language models on compositional reasoning tasks.

Method	LLM	Params	SugarCrepe
MiniGemini-2B	Gemma-2B	~2B	67.0
Imp-2B	Qwen1.5-1.8B	~2B	71.0
Bunny-2B	Qwen1.5-1.8B	~2B	68.6
LLaVA-KD-2B	Qwen1.5-1.8B	~2B	75.3
Ours	Qwen1.5-1.8B	~2B	83.6

**Table 3:** Comparison of different distillation methods and regional intervention strategies.

Method	LLM	GQA	TextVQA	MME	Sugarcrepe
KD-2B	Qwen1.5-1.8B	62.3	53.4	68.9	75.3
LLaVADI-2B	Qwen1.5-1.8B	58.7	49.4	63.8	76.9
MoD-2B	Qwen1.5-1.8B	58.8	52.7	63.4	76.9
GBIS-2B	Qwen1.5-1.8B	62.7	56.3	69.8	81.7
CompoDistill-2B	Qwen1.5-1.8B	62.2	56.4	70.1	82.9
Ours	Qwen1.5-1.8B	63.3	57.3	70.9	83.6

**Table 4:** Ablation study of the CEAF and MAD modules.

CEAF Module	MAD Module			GQA	TextVQA	MME	SugarCrepe
	Global	Top-K	Ranking				
				57.8	50.1	62.5	76.3
**✓**				58.6	52.0	64.1	81.7
**✓**	**✓**			59.0	53.7	65.3	82.1
**✓**		**✓**		59.5	54.1	64.5	82.5
**✓**			**✓**	59.7	53.4	64.9	82.0
	**✓**			61.0	55.8	68.0	79.4
**✓**	**✓**			63.3	57.3	70.9	83.6

✓ indicates that the corresponding module or loss component is enabled; blank entries indicate that it is disabled.

**Table 5:** Performance comparison with different teacher and student model sizes.

Student	Teacher	VQA Avg	SugarCrepe
Qwen1.5-0.5B	Qwen1.5-4B	58.5	71.5
Qwen1.5-1.8B	Qwen1.5-4B	64.9	83.5
Qwen1.5-1.8B	Qwen2.5-7B	65.7	84.5

**Table 6:** Detection results of various methods on VisDrone.

Method	mAP50	mAP(0.5:0.95)	Params
*Traditional SOTA UAV Models*			
RetinaNet	22.1	16.6	19.8M
Faster-RCNN	33.5	19.3	41.2M
Yolov5-s	37.9	22.7	9.1M
Yolov7-tiny	35.4	18.9	6.0M
Yolov8-n	31.9	18.4	3.0M
Yolov10-n	31.7	18.4	2.71M
*VLMs*			
YOLO-World	55.3	-	110M
LAE-DINO	56.4	-	-
OURS	62.0	39.0	2B

**Table 7:** Comparison of detection performance and statistical uncertainty.

Comparison	mAP50 ± Std	mAP (0.5:0.95) ± Std	95%CI (mAP)	95%CI (mAP50)
Yolov5-s	37.9	22.7		
YOLO-World	55.3	-		
OURS	62.0 ± 0.7	39.0 ± 0.36	mAP [38.10, 39.90];	mAP50 [60.26, 63.74]

## Data Availability

Data Availability Statement: This study used publicly available training and evaluation datasets. The training datasets, LLaVA-Pretrain-558K and LLaVA-v1.5-Mix-665K, are available at https://huggingface.co/datasets/liuhaotian/LLaVA-Pretrain (accessed on 28 October 2026) and https://huggingface.co/datasets/liuhaotian/LLaVA-Instruct-150K/blob/main/llava_v1_5_mix665k.json (accessed on 28 October 2026). The evaluation datasets are available from the following sources: GQA (https://cs.stanford.edu/people/dorarad/gqa/, accessed on 28 October 2026); TextVQA (https://textvqa.org/, accessed on 28 October 2026); MME (https://mme-benchmark.github.io/home_page.html, accessed on 28 October 2026); SugarCrepe (https://github.com/RAIVNLab/sugar-crepe, accessed on 28 October 2026); VizWiz-VQA (https://vizwiz.org/tasks-and-datasets/vqa/, accessed on 28 October 2026); and VisDrone2019-DET (https://github.com/VisDrone/VisDrone-Dataset, accessed on 28 October 2026).
